# Correlation between sarcopenia and left ventricular myocardial mass in chronic heart failure patients

**DOI:** 10.1002/agm2.12111

**Published:** 2020-06-18

**Authors:** Mei Wang, Song Hu, Furong Zhang, Jia Liu, Yongjun Mao

**Affiliations:** ^1^ Department of Geriatric Medicine The Affiliated Hospital of Qingdao University Qingdao China; ^2^ Department of Internal Medicine Laixi People’s Hospital Laixi China

**Keywords:** chronic heart failure, myocardial mass, sarcopenia

## Abstract

Sarcopenia is defined as a progressive age‐related syndrome of reduced whole‐body muscle mass, muscle strength, and muscle function. Patients with heart failure (HF) and sarcopenia are inclined to have decreased muscle strength. The current research suggests that skeletal muscle changes are the main manifestations of sarcopenia, but myocardium is not mentioned as skeletal muscle. The myocardium changes significantly with the progression of HF. Measuring myocardial quality has become an important and accessible way to assess HF by detecting changes in myocardial quality in patients with sarcopenia. Due to its economical, simple, and effective advantages in measuring myocardial quality, real‐time three‐dimensional echocardiography provides a convenient and reliable method for clinical diagnosis of HF and sarcopenia. This will be conducive to the screening of sarcopenia in people with chronic HF to better guide clinical treatments. This review describes the definition and diagnostic criteria of sarcopenia, the characteristics of HF with sarcopenia, and the related study of myocardial quality.

## 1 INTRODUCTION

The aging‐population problem in China is becoming more and more obvious. Sarcopenia, which is related to various adverse health effects, is a syndrome of senility and has been extensively studied in recent years. Meanwhile, the relationship between sarcopenia and heart failure (HF) also receives attention. However, because of the complexity of cardiac changes, such as cardiac hypertrophy and hypotrophy, the correlation between sarcopenia and myocardial mass has currently received minimal attention. This review introduces sarcopenia, HF, and myocardial mass, with a focus on the correlation between sarcopenia and myocardial mass.

## 2 SARCOPENIA

Sarcopenia is defined as a progressive syndrome that reduces whole‐body muscle mass, muscle strength, and muscle function.^1^ In the elderly, due to the advancement of age, the function and strength of muscles may be reduced or weakened, affecting patients’ ability to be active. Currently, skeletal muscle mass index (muscle mass), grip strength (muscle strength), and usual gait speed (muscle function) are measured to diagnose sarcopenia according to the Asian Working Group for Sarcopenia.^2^ Evidently, the diagnosis and research of sarcopenia are mainly limited to skeletal muscle.

## 3 SARCOPENIA‐RELATED HF AND REDUCED MYOCARDIAL MASS

It is well established that sarcopenia is closely related to chronic HF (CHF). The prevalence of sarcopenia in elderly patients with CHF is higher than that in patients without a condition of CHF. CHF may cause sarcopenia through common pathogenetic pathways, including inflammation, hormonal changes (such as insulin‐like growth factor‐1, angiotensin, sex hormone, myostatin), physical inactivity, low ventricular ejection fraction, malnutrition, and oxidative stress.^3^ In contrast, sarcopenia, or a reduction of muscle strength and mass, may favor the development of CHF via different mechanisms. Recent studies confirm that CHF is characterized by the presence of several modifications, including those related to hormones (including insulin, growth hormone, prolactin, thyroid hormones, catecholamines, and corticosteroids), apoptosis, physical inactivity, low muscle blood flow, and endothelial dysfunction.^4^ These HF‐related factors may be activated by the development of sarcopenia. Moreover, patients with CHF are prone to displaying decreased exercise tolerance and exercise ability. Consequently, this accelerates the development of sarcopenia.

As is well known, changes in the myocardial cells and myocardial mass are important clinical changes of CHF. For instance, atrophy of myocardial cells and reduction of myocardial mass are essential stages of HF. Due to the strong correlation between sarcopenia and CHF, the effect of sarcopenia on myocardial mass is highly significant. Furthermore, both cardiac and skeletal muscle is striated muscle.^3^ Consequently, sarcopenia, whose main manifestation is skeletal muscle, is also related to myocardial mass.

## 4 IMPLICATIONS OF HAVING BOTH SARCOPENIA AND REDUCED MYOCARDIAL MASS OR HF

Generally, a series of symptoms, such as dyspnea, low physical ability, frailty, and reduced ejection fraction, would appear in the early stage of CHF, with some maybe appearing latter. Now we can infer that the symptoms above are not only caused by decreases of cardiac function, but also sarcopenia. Sarcopenia can result in low muscle mass and decreased muscle function of whole‐body muscle (for example limb muscle, respiratory muscle, and myocardial muscle), leading to those symptoms. Compared with healthy people or patients with a single disease, once complicated with sarcopenia and HF or reduced myocardial function, patients are more prone to disease progression and poorer prognosis.

## 5 MECHANISMS OF REDUCED MYOCARDIAL MASS IN SARCOPENIA

**Figure 1:**
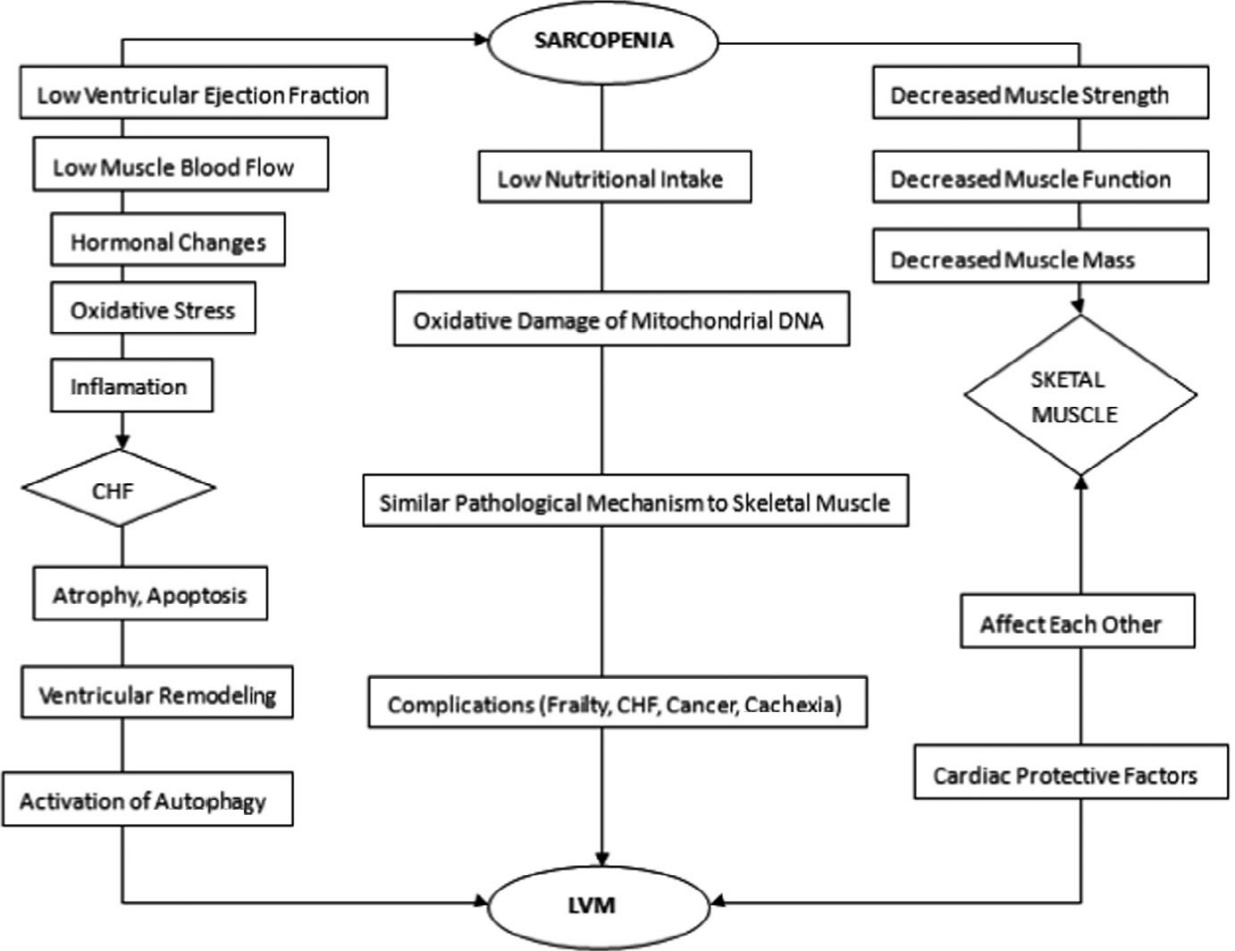
Correlation between sarcopenia and left ventricular mass (LVM). CHF, chronic heart failure.

The relationship between sarcopenia and myocardial mass deserves our attention. Based on previous research and theory, this review puts forward the following opinions about mechanisms of reduced myocardial mass in sarcopenia, as shown in Figure 1:

1. Sarcopenia affects myocardial mass by acting on CHF. Like the factors mentioned above, CHF patients suffer from decreased cardiac function and cardiac hypertrophy, which leads to ventricular remodeling and CHF. With the aggravation of the disease, the myocardium is gradually affected by hypoxia and necrosis. The fiber cells replacing the injured myocardium further contribute to decreased myocardial mass.^5^ Furthermore, in CHF, impaired autophagy (which may lead to increased oxidative stress and reduced adenosine triphosphate production) ultimately contributes to age‐related skeletal muscle loss.^4^ Recent studies confirm that the endothelial function in CHF patients with sarcopenia is greatly reduced.^5^ In the elderly, sarcopenia is closely associated with atherosclerosis. As a result, sarcopenia may contribute to the formation of CHF lesions. In fact, recent research has proposed the term "cardiac skeletal myopathy" to clarify the mechanism.^6^ It is an important cause of decreased physical fitness and poor cardiopulmonary function in elderly CHF patients.

2. Like skeletal muscle, myocardial mass is one of the direct targets of sarcopenia. At first, similar to the skeletal muscle, the myocardium is also a striated muscle.^5^ Therefore, the corresponding pathological mechanism may also be similar. Sarcopenia is characterized by atrophy of the type II muscle fibers and a progressive denervation and re‐innervation process that results in the loss of a motor unit.^2^ From a histopathological perspective, muscle dystrophy is majorly manifested as atrophy and apoptosis of skeletal muscle fibers and continuous reduction of organelles, such as mitochondria dysfunction.^7^ In cardiomyocytes, angiotensin II may lead to apoptosis, ubiquitin‐proteasome system activation, continuous sympathetic nerve activity, and excessive oxidative stress by modifying the insulin‐like growth factor‐1 signal. This may cause mitochondrial damage, muscle protein degradation, and decreased appetite, eventually leading to muscle atrophy.^8^ These findings were confirmed in animal models. In addition, animal studies suggest that the advancement of age results in the oxidative damage of mitochondrial DNA and an increase in intercellular connective tissue within cardiomyocytes.^9^ These cellular changes are linked to sarcopenia. Secondly, the infiltration of fat and connective tissue is another important element of reduced muscle mass. As adipose tissue is constantly deposited between muscle fibers, the number of muscle satellite cells continues to decrease, and muscle function declines further.^3^ In addition, sarcopenia can also weaken left ventricular mass (LVM) through some common pathways, including muscle tissue remodeling, complications (eg, frailty and CHF), low nutritional intake, and lack of activity.^3^, ^5^ Finally, the activation of ubiquitin and increased protein degradation can lead to a reduction in muscle protein synthesis.^9^ Therefore, we conclude that sarcopenia could lead to a decrease in LVM even without CHF.

3. Skeletal muscle may affect the myocardial mass directly. In animal model experiments, it is observed that a class of substances, such as Akt protein kinase B, is secreted by the skeletal muscle.^10^ These substances are known as cardiac protective factors and may reduce cardiac injury.^3^ However, recent evidence suggests that pathological changes of skeletal muscle in patients with sarcopenia may reduce the protective effects of cardiac protective factors.^5^ Research shows that LVM atrophy arises in conjunction with losses of skeletal muscle.^3^, ^5^ Sarcopenia may lead to a decrease in the peripheral skeletal muscle, a decrease in myocardial muscle mass, and accelerated myocardial hypertrophy, thereby worsening CHF. As a result, sarcopenia is closely related to myocardial mass.

## 6 ASSESSMENT OF LOW MYOCARDIAL MASS

It is important to examine the myocardium for the assessment and diagnosis of sarcopenia. In myocardial remodeling, changes in the LVM and the left ventricular mass index (LVMI) are particularly evident. LVM is an indicator of heart disease and is closely associated with cardiac function. LVM also plays an important role in the diagnosis of cardiovascular diseases and HF. Accurate measurement of LVM is important for the evaluation of cardiovascular diseases and HF. However, no detection method is currently available. Real‐time three‐dimensional echocardiography is a non‐invasive, inexpensive, and easy method. It has gained clinical recognition and has an increasingly critical role in the detection of cardiac function, ejection fraction, ventricular morphology, and myocardial contractile function. It is also gaining recognition in the evaluation of left ventricular myocardial mass and cardiac function in cardiovascular diseases.^11^ Devereux’s ventricular weight correction formula^11^ may be used to measure LVM and LVMI. Left ventricular endothelium (LVEDD), interventricular septal thickness (IVS), and left ventricular posterior wall thickness (LVPW) at ventricular end‐diastolic may be measured via a real‐time three‐dimensional ultrasound. LVM is calculated using Devereux’s empirical formula as follows: LVM = 1.05 × [(LVEDD + IVS + LVPW)^3 ^− (LVEDD)^3^] − 13.6.^11^ The average is obtained by calculating the value three times in succession. Left ventricular myocardial mass index: LVMI (g/m^2^) = LVM/BSA, that is, each LVM value obtained is divided by body surface area (BSA) to determine the LVMI.

## 7 CONCLUSION

In summary, this review summarizes the findings of sarcopenia, HF, and myocardial mass from epidemiology, pathology, clinical reports, and other research. In patients with HF, sarcopenia is not only manifested as skeletal muscle changes, but also as myocardial transformations. In addition, this review infers from previous research that myocardial quality is modified in patients with sarcopenia. In patients with CHF and sarcopenia, peripheral muscle decline is accompanied by a decrease in myocardial mass. Using cardiac mass and myocardial quality as indices to evaluate sarcopenia may prove effective for the screening of sarcopenia in people with CHF. Additionally, it may guide clinical treatments.

## CONFLICTS OF INTEREST

Nothing to disclose.

## AUTHOR CONTRIBUTIONS

Writing of paper: All authors. Mei Wang: Design, literature review, and coordination. Jia Liu: Design and data collection. Furong Zhang and Song Hu: Data collection and cleansing, review of medical records, and statistical analysis. Professor Yongjun Mao: Data analysis, review of medical notes, and statistical analysis.
